# A self-supporting bimetallic Au@Pt core-shell nanoparticle electrocatalyst for the synergistic enhancement of methanol oxidation

**DOI:** 10.1038/s41598-017-06639-5

**Published:** 2017-07-24

**Authors:** Changhui Tan, Yinghui Sun, Jianzhong Zheng, Dan Wang, Ziyang Li, Huajie Zeng, Jun Guo, Liqiang Jing, Lin Jiang

**Affiliations:** 1 Institute of Functional Nano & Soft Materials Laboratory (FUNSOM), Jiangsu Key Laboratory for Carbon-Based Functional Materials & Devices, Soochow University, Suzhou, 215123 China; 2 College of Chemistry and Environment, Fujian Province Key Laboratory of Morden Analytical Science and Separation Technology, Minnan Normal University, Zhangzhou, 363000 P. R. China; 3 Key Laboratory of Functional Inorganic Materials Chemistry (Heilongjiang University), Ministry of Education, School of Chemistry and Materials Science, Harbin, 150080 P. R. China; 4 Soochow Institute for Energy and Materials InnovationS, College of Physics, Optoelectronics and Energy & Collaborative Innovation Center of Suzhou Nano Science and Technology, Soochow University, Suzhou, 215006 China; 5 Testing and Analysis Center, Soochow University, Suzhou, 215123 China

## Abstract

The morphology of Pt−Au bimetal nanostructures plays an important role in enhancing the catalytic capability, catalytic stability and utilization efficiency of the platinum. We designed and successfully prepared Au@Pt nanoparticles (NPs) through an economical, surfactant-free and efficient method of seed-mediated growth. The Au@Pt NPs displayed electrochemical performances superior to those of commercial Pt/C catalysts because their agglomeration was prevented and exhibited better long-term stability with respect to methanol oxidation in acidic media by efficiently removing intermediates. Among the obtained Au@Pt NPs, Au_90_@Pt_10_ NPs exhibited the most significantly enhanced catalytic performance for the methanol oxidation reaction (MOR). Their mass and electrochemically active surface area (ECSA)-normalized current densities are approximately 3.9 and 4.6 times higher than those of commercial Pt/C catalysts, respectively. The oxidation current densities of the Au_90_@Pt_10_ NPs are approximately 1.8 times higher than those of commercial Pt/C catalysts after 4000 s of continuous measurement because the small Pt NPs grown on the surface of the Au_90_@Pt_10_ NPs were effectively stabilized by the Au metal support. This approach may be a facile method for the synthesis of self-supported bimetallic nanostructures, which is of great significance for the development of high performance electrocatalysts and sensors.

## Introduction

In the face of the oil and other fossil fuel crises, the development of clean and sustainable energy sources has become an increasingly important concern. Direct methanol fuel cells (DMFCs) are the desired power source for electronic devices because of the high energy density of methanol and its biological renewability, high energy conversion efficiency, low cost and very low environmental pollution^1–4^. Platinum (Pt) is generally considered the most efficient and common catalyst for both anodes and cathodes for DMFCs^5–16^. However, first, the relative scarcity and high cost of Pt hinder the large-scale manufacture and development of DMFCs^17–19^. Second, the stability and activity of catalysts are major challenges for widespread applications of DMFCs. Other metals (e.g., Au, Ag, Pd, Ni, Fe, Cu) have been introduced into Pt to form Pt-based nanostructures, which not only minimize the usage of Pt but also improve the overall electrocatalytic performance^20–28^. Therefore, the design and synthesis of Pt-based nanostructures with enhanced catalytic capability and utilization efficiency of Pt is an ongoing goal of chemists.

Bimetallic systems with synergistic effects that are tunable through variations of composition and structure have many potential applications in the methanol oxidation reaction (MOR)^29–31^. To enhance the mass activity of Pt-based catalysts, one of the most typical strategies is to incorporate non-Pt (M) metals into the Pt nanoparticles (NPs) by alloying technology or by using core-shell composite bimetallic nanostructures^32–40^. In core-shell bimetallic nanostructures, the M metal is the core of the NPs, and Pt metal is located on the surface the M core. Thus, because of the interaction between Pt and M, not only is the catalytic activity greatly enhanced but also the usage of Pt metal is notably decreased^31, 41^. To date, core-shell nanostructures with enhanced performances as catalysts for the methanol oxidation reaction (MOR) have been observed^42–53^. Gold (Au) is a unique metal since it is inert in the bulk state but has high catalytic activity for many reactions at the nanoscale^54–57^. The special properties of Au at the nanoscale have also been found to grant unparalleled electrocatalytic activity for CO oxidation^58–60^. Consequently, bimetallic Pt–Au nanocatalysts are expected to exhibit synergistic catalytic effects toward the MOR. First, Pt in the shell of Au-Pt core-shell nanostructures grows on the surface of Au and shares the same crystalline directions as the Au support^61, 62^. Second, Au is inert in acid electrolytes, demonstrating high chemical stability and durability as a catalyst component^63^. Third, the synergistic effect from the Au and Pt interaction alters the structure of the Pt electronic bands by changing the surface adsorption force^31, 64–66^, which contributes to a significant enhancement of both electrocatalytic activity and stability that could grant improved CO tolerance to Pt catalysts in the MOR. Various approaches have been developed to synthesize bimetallic Au-Pt core-shell nanostructures, including solvothermal, electrodeposition or template methods. However, these preparation methods usually require high temperatures, surfactants, electrical energy and/or sacrificial templates^42, 44–47, 49, 50^, which create complicated, power-wasting and time-consuming processes. Moreover, the surfactant-stabilizing nanostructures interfere with the catalytic performance of Pt in the MOR^67^.

Herein, we demonstrate a facile and surfactant-free approach for the synthesis of spiny Au@Pt core-shell nanoparticles. The reason for choosing spiny Au NPs as cores for Pt growth is that spiny Au NPs have a large specific surface area because of the high density of spines on the Au NP surface, and the small Pt NPs can be uniformly loaded on the naked surface of spiny Au NPs without surfactant capping. Thus, both electrocatalytic stability and activity are greatly enhanced in methanol oxidation in acidic conditions. With the optimized Pt amount loading on the spiny Au NPs, the Au_90_@Pt_10_ NPs exhibited enhanced catalytic performance, with their mass and electrochemically active surface area (ECSA)-normalized current densities being approximately 3.9 and 4.6 times higher than those of commercial Pt/C catalysts, respectively. Spiny Au NPs play an important role in decreasing precious Pt content and increasing stability and activity during methanol oxidation and signify the importance of supporting matrices in Pt-based catalysts. We expect that this method will serve as a facile and effective approach to fabricate bimetallic nanostructures for further applications in DMFCs with high efficiency.

## Results

### Morphological Control of the self-supporting Au@Pt NPs

**Figure 1:**
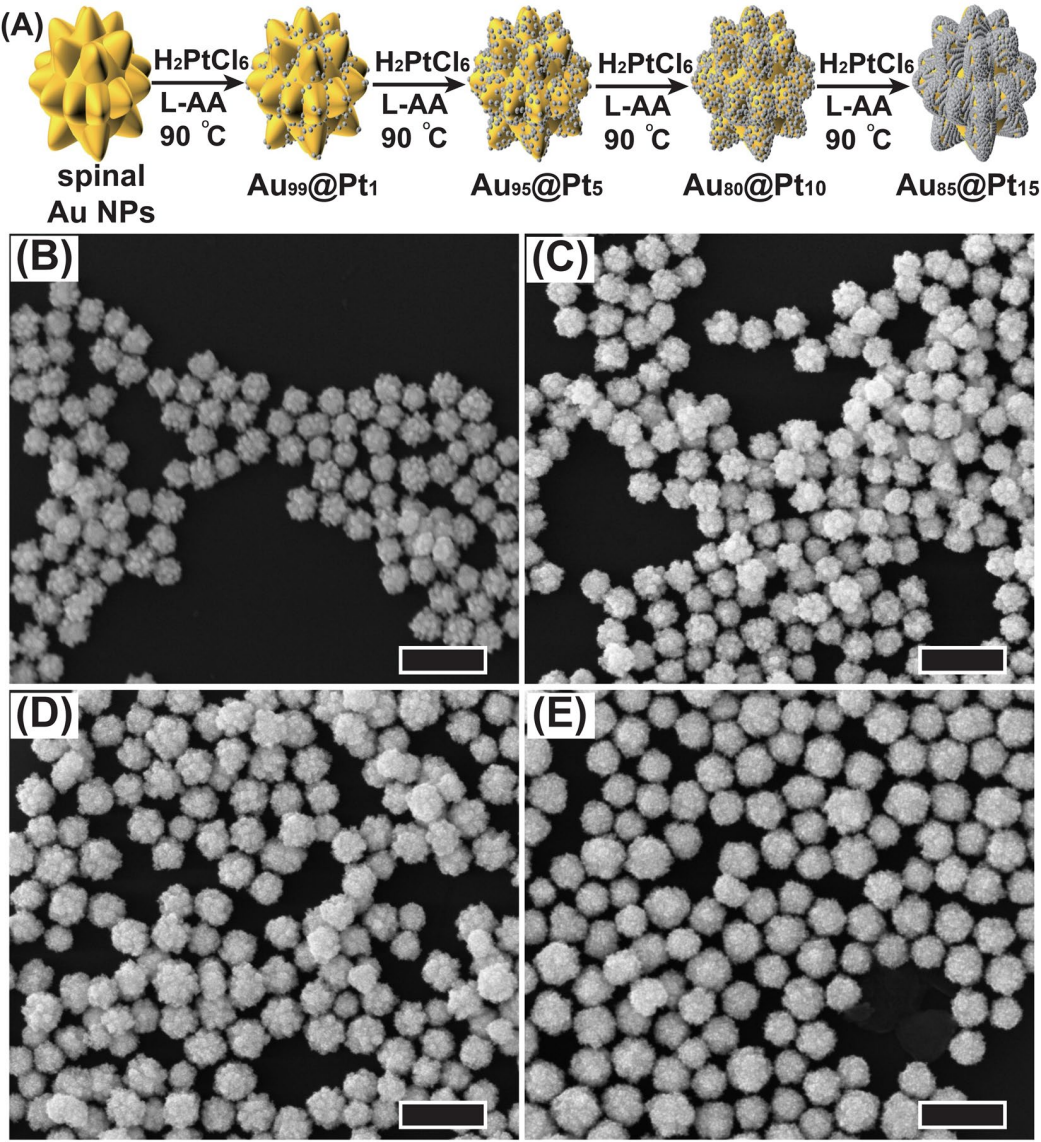
(**A**) Reaction scheme showing the morphological and structural changes involved in the fabrication of Au@Pt NPs with different Pt ratios. SEM images of Au@Pt NPs with different Pt source amount-modulated spiny Au NPs. The scale bar at the lower right corner indicates 200 nm. (**B**) Au_99_@Pt_1_ NPs (**C**) Au_95_@Pt_5_ NPs (**D**) Au_90_@Pt_10_ NPs (**E**) Au_85_@Pt_15_ NPs.

The preparation procedure of the self-supporting Au@Pt NPs is shown in Fig. 1A. Spiny Au NPs were prepared and used as seeds to synthesize Au@Pt NPs, which is a process of chemical growth and an element-replacement reaction. The synthesized spiny Au NPs were dispersed into an aqueous solution containing chloroplatinic acid. According to the literature, it is much easier and faster to reduce Pt(II) ions than Pt(IV) ions^68, 69^. Therefore, the Pt(IV) ion was chosen in this study to obtain a slow reduction speed. Ascorbic acid was used as a weak reductant to reduce Pt(IV) ion^70^. In this process, the spiny Au NPs provided a nucleation center, and the Pt atoms were uniformly and slowly reduced on the surface of the spiny Au NPs. The reduction of the Pt(IV) ions more preferentially occurred on the surface of the spiny Au NPs than in the solution^61^. As the reaction proceeded, more Pt ions were reduced into atoms by L-ascorbic acid and grew into small Pt NPs. These Pt NPs were uniformly loaded on the surface and formed a strong connection with the spiny Au NPs. With the chemical growth of these small Pt NPs, they connected to each other to form a shell on the surface of the spiny Au NPs as the amount of Pt increased. The amount of Pt can be regulated by adding specific volumes of Pt precursors in the synthesis. Au@Pt NPs with different Pt ratios (Au_99_@Pt_1_, Au_95_@Pt_5_, Au_90_@Pt_10_, Au_85_@Pt_15_) were obtained by changing the injected amount of chloroplatinic acid (H_2_PtCl_6_) and were determined by inductively coupled plasma atomic emission spectroscopy (ICP-AES) (see Supplementary Table S1).

The micromorphology of the Au@Pt NPs was evaluated by scanning electron microscopy (SEM) as shown in Fig. 1B–E. The spiny Au NPs are evenly dispersed and have little agglomeration, with uniform shapes and an average diameter of approximately 80 nm, which provides good conditions for the direct growth of the Pt NPs (see Supplementary Figure S1). After being decorated with small Pt NPs, the Au@Pt NPs retained a morphology similar to that of the spiny Au NPs. With a lower coated quantity of platinum, the morphology of the Au_99_@Pt_1_ NPs Pt appears almost the same as that of the spiny Au NPs (Fig. 1B), where it is difficult to observe any clear small Pt NPs on the surface of the spiny Au NPs. As shown in Fig. 1C–E, with the increase in the amount of platinum from the Au_95_@Pt_5_ NPs to the Au_90_@Pt_10_ NPs, Pt NPs were clearly observed to be distributed on the surfaces of the spiny Au NPs. If the coated quantity of platinum was excessive, so many Pt NPs were coated onto the surfaces of the spiny Au NPs that the Pt NPs were overgrown on the surface, creating the appearance that the spines were getting shorter, as shown in Fig. 1E.

**Figure 2:**
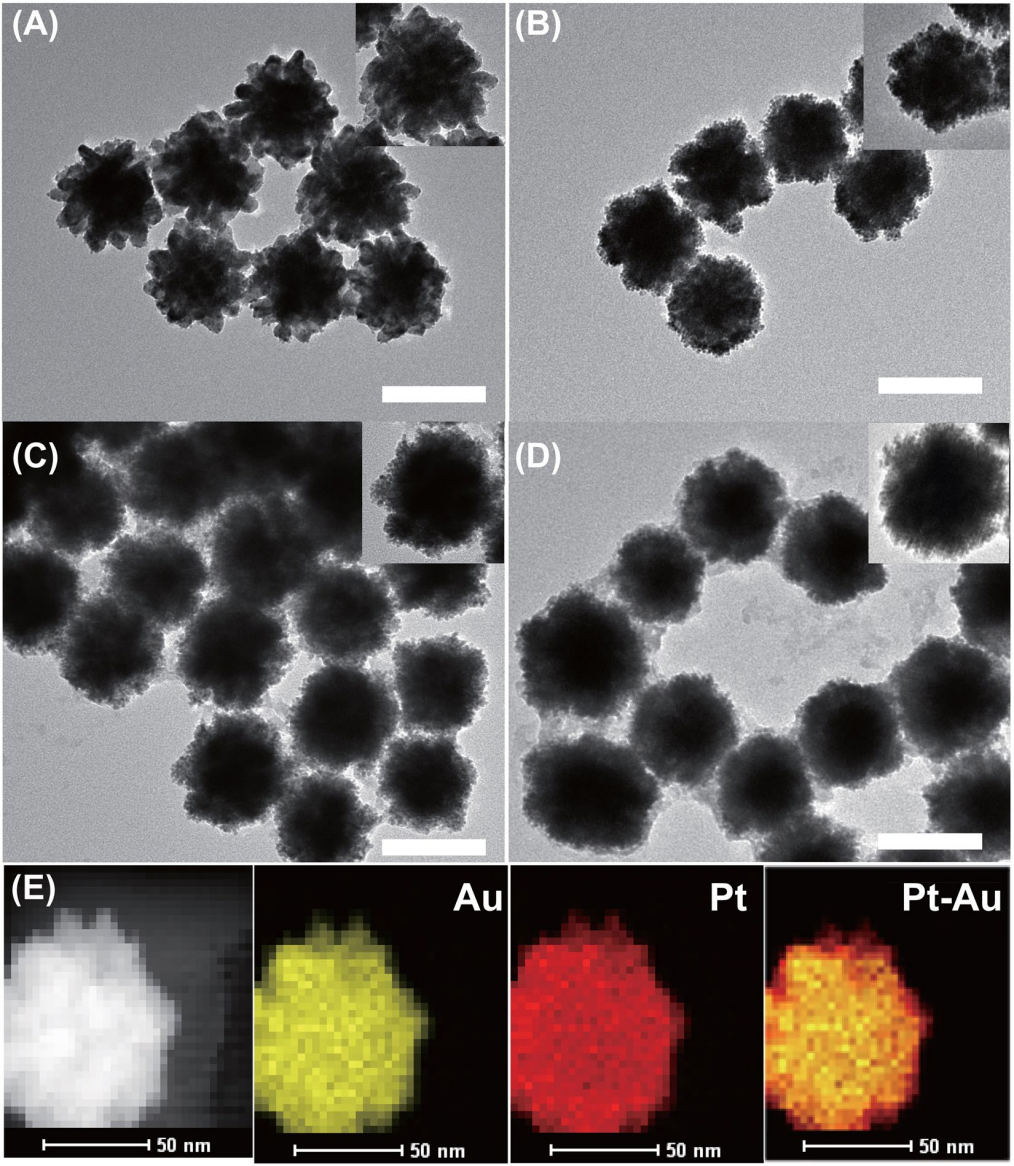
TEM images of different Pt source amount-modulated spiny Au NPs. The insets are the single-nanoparticle views of the four types of Au@Pt NPs. (**A**) Au_99_@Pt_1_ NPs (**B**) Au_95_@Pt_5_ NPs (**C**) Au_90_@Pt_10_ NPs (**D**) Au_85_@Pt_15_ NPs. Scale bar: 100 nm. (**E**) HAADF-STEM image and elemental mappings of Au_90_@Pt_10_ NPs.

Transmission electron microscopy (TEM) was performed to further analyze the structure and components of the Au@Pt NPs. As shown in Fig. 2, Pt nanoparticles were uniformly distributed on the surfaces of the spiny Au NPs. Figure 2A shows that Au_99_@Pt_1_ NPs, prepared at a low concentration of H_2_PtCl_6_ in solution, had only a small quantity of Pt NPs dispersed on the tips of the spiny Au NPs. When the concentration of H_2_PtCl_6_ was increased, the increase in Pt NPs on the surfaces and tips of the spiny Au NPs can clearly be observed in Fig. 2B and C. However, in the Au_85_@Pt_15_ NPs, the surfaces and tips of the spiny Au NPs were completely coated with platinum nanoparticles, forming a continuous core-shell structure as illustrated in Fig. 2D, which led to nanospheres from the spiny NPs. This result indicated that the concentration of H_2_PtCl_6_ plays an important role in the formation of Pt nanoparticles and that the morphology of the Au@Pt NPs could be tuned by varying the concentration of H_2_PtCl_6_.

The distributions of the metallic elements in the Au@Pt NPs were important to the catalytic ability; they were analyzed by means of high-angle annular dark-field scanning transmission electron microscopy (HAADF-STEM) (Fig. 2E). The merged mapping patterns provided clear evidence for the elemental distribution of Au and Pt in the Au_90_@Pt_10_ NPs, which further indicated the core-shell structure of the Au_90_@Pt_10_ NPs. Accordingly, elemental mapping of Au (yellow color) demonstrated that Au is distributed in the interior; Pt (red color) is decorated on the surface of the spiny Au NPs, and Pt seemed to be homogenously distributed over the entire surface of the Au core at this concentration.

**Figure 3:**
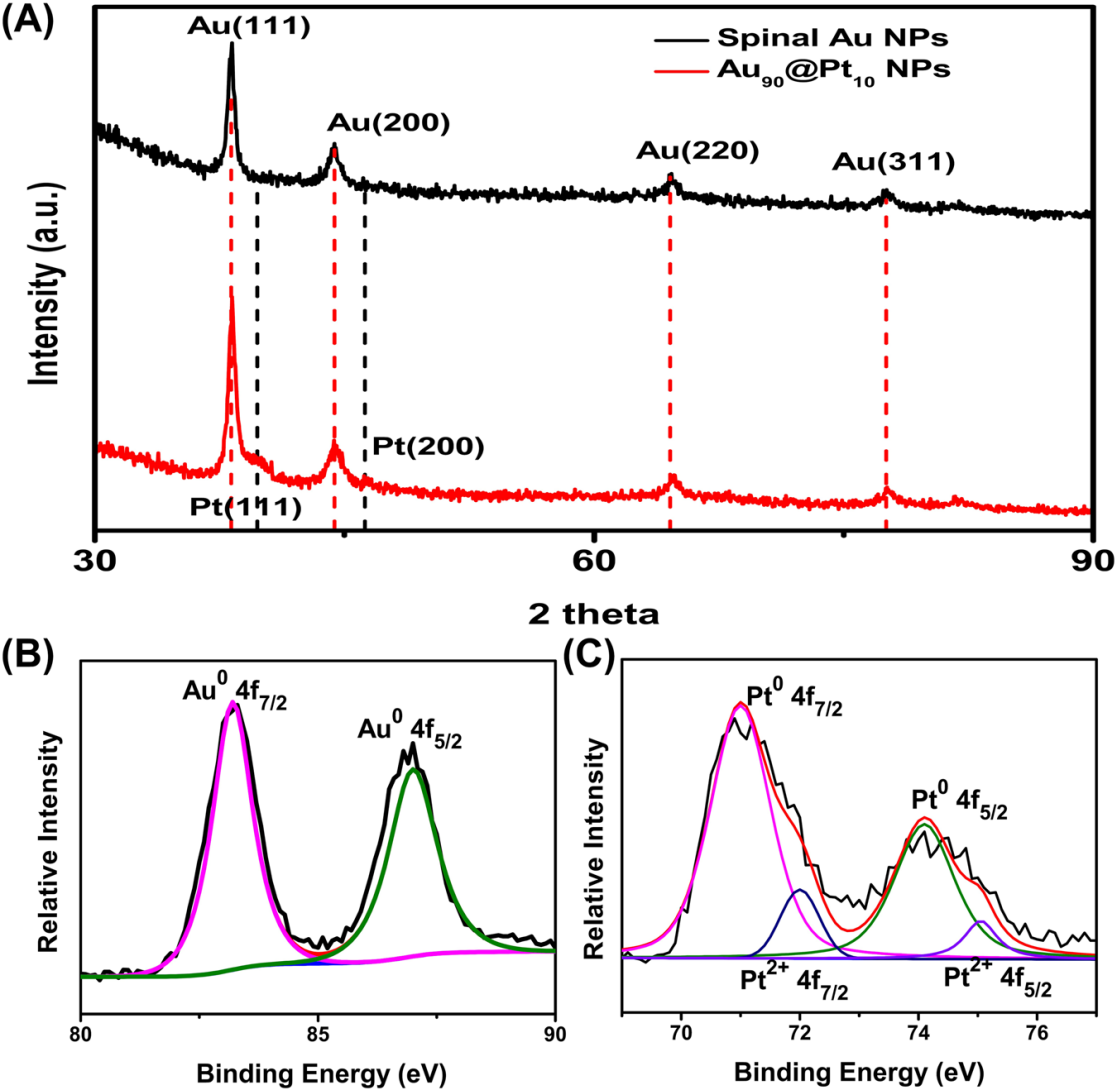
(**A**) XRD pattern of the Au_90_@Pt_10_ NPs. (**B**) Au 4 f and (**C**) Pt 4 f XPS spectra of the Au_90_@Pt_10_ NPs.

The composition of the Au_90_@Pt_10_ NPs was further investigated by X-ray diffraction (XRD), shown in Fig. 3A. The diffraction peaks of the Au_90_@Pt_10_ NPs can be categorized into two sets. The peaks at two theta values of 38°, 44°, 64° and 77° can be indexed to the (111), (200), (220) and (311) crystal planes of face-centered cubic Au (JCPDS card No. 4-784), respectively, which are similar to the diffraction peaks of the spiny Au NPs. The other set of peaks, at two theta values of 39° and 46°, can be ascribed to the (111) and (200) crystal planes of face-centered cubic Pt (JCPDS card No. 4-802). The crystal structures of the spiny Au NPs and Au_90_@Pt_10_ NPs were further characterized by means of high-resolution TEM (HRTEM). The lattice spacings of the spiny Au and Pt NPs (see Supplementary Figure S2) are 0.235 and 0.226 nm, which match well with the d-spacings of the Au (111) and Pt (111) planes (0.235 nm for Au, 0.226 nm for Pt), respectively. These results indicate that Au and Pt nanoparticles exist individually in the Au_90_@Pt_10_ NPs.

To investigate the interaction of Au and Pt and the surface oxidation state, typical X-ray photo electron spectroscopy (XPS) was employed, and the spectra of the Au_90_@Pt_10_ NPs are shown in Fig. 3. Figure 3B presents the Au 4 f core level spectrum for the Au_90_@Pt_10_ NPs, in which the peaks at 83.6 and 87.2 eV can be attributed to Au 4 f_7/2_ and Au 4f_5/2_ of Au^0^. In the Pt 4f core level spectrum of the Au_90_@Pt_10_ NPs (Fig. 3C), two peaks at 71.1 and 74.40 eV can be assigned to Pt 4f_7/2_ and Pt 4f_5/2_ of Pt^0^. Additionally, the peaks at 71.9 and 75.2 eV correspond to Pt^2+^ species^71^. According to the high (or highest) Pt^0^/Pt^2+^ intensity ratio, it can be inferred that the Pt^0^ species was dominant. Compared to the standard data of pure atomic Au 4 f (84.0 and 87.7 eV), and Pt 4 f (70.9 and 74.2 eV)^36, 61^, the binding energies of Au 4 f were shifted to lower values, and those of Pt 4 f were shifted to higher values. The decrease in the binding energies of Au 4 f and the increase in those of Pt 4 f suggest that Au and Pt in the bimetallic Au_90_@Pt_10_ NPs could possess a charge transfer phenomenon. This may contribute to a potential increase in the stability of the Au@Pt NP electrocatalyst.

### Electrocatalytic Performances of self-supporting Au@Pt NPs toward the MOR

**Figure 4:**
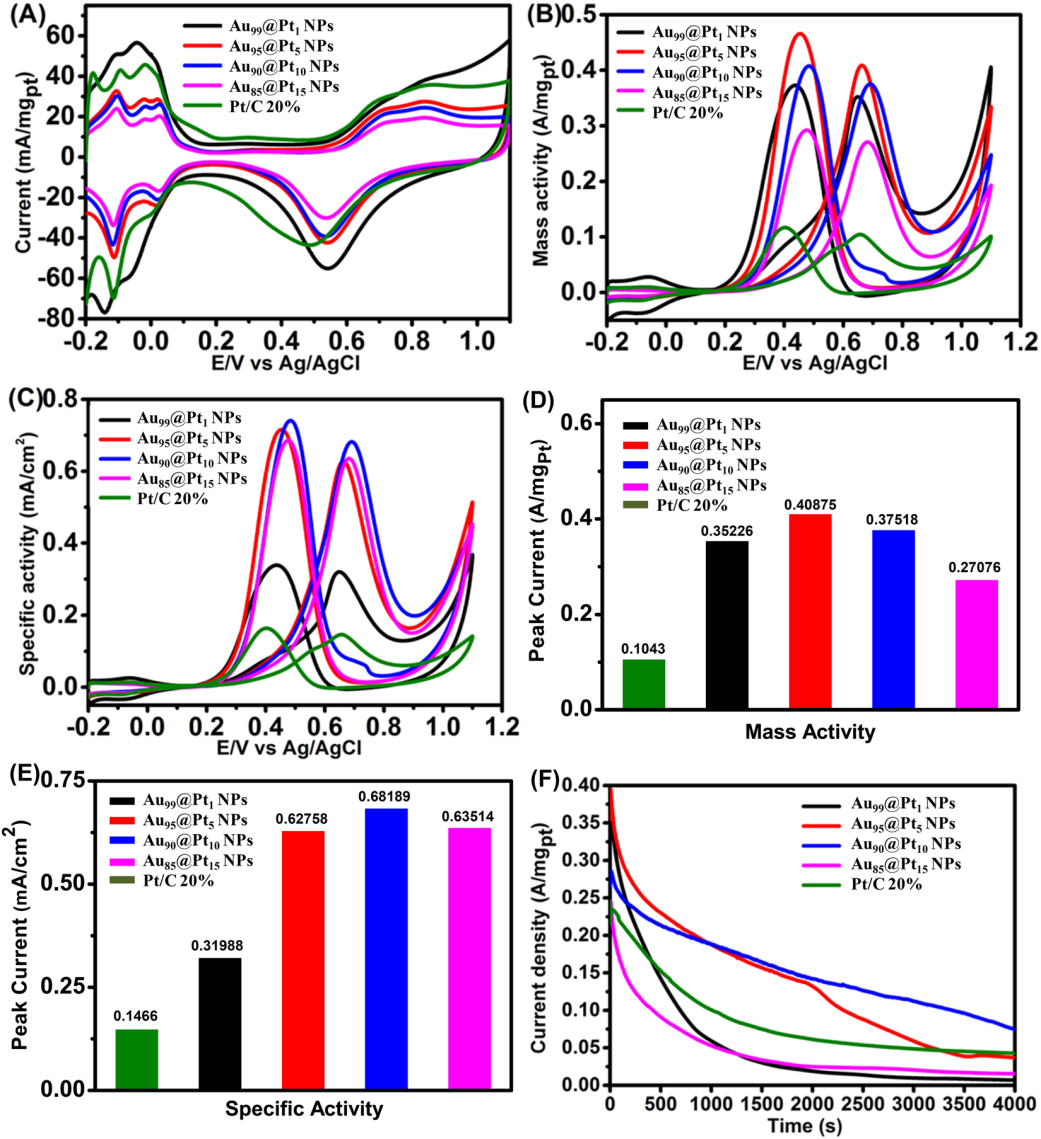
(**A**) CV curves of the four types of Au@Pt NP catalyst and commercial Pt/C catalyst in 0.5 M N_2_-saturated sulfuric acid solution. (**B**) Mass activity CVs and (**C**) specific activity CVs of the four types of Au@Pt NP catalyst and the commercial Pt/C catalyst in 0.5 M H_2_SO_4_ + 0.5 M CH_3_OH. Histograms of the peak current of the four types of Au@Pt NP catalyst and the commercial Pt/C catalyst: (**D**) Mass activity, (**E**) Specific activity. (**F**) Chronoamperometric curves of the four types of Au@Pt NP catalyst and the commercial Pt/C catalyst.

Spiny Au Nps without Pt do not present any electrochemical activity in sulfuric acid and with the addition of ethanol (see Supplementary Figure S3). The electrocatalytic performances of the as-prepared self-supporting Au@Pt NPs with different Pt contents were studied by cyclic voltammetry (CV) at room temperature in nitrogen-purged solutions at a sweep rate of 50 mV/s. The catalytic properties of commercial 20% Pt/C were also tested for comparison. Figure 4A shows that the CV curves of the five catalysts exhibit typical hydrogen adsorption/desorption peaks at potentials between −0.2 and 0.15 V and oxygen regions in the range of 0.6−1.0 V. The electrochemically active surface area (ECSA) was calculated by measuring the area of the desorption region between −0.2 and 0.15 V after double-layer correction. The ECSAs of the Au_99_@Pt_1_ NPs, Au_95_@Pt_5_ NPs, Au_90_@Pt_10_ NPs, and Au_85_@Pt_15_ NPs are 110.12, 65.11, 55.02 and 42.63 m^2^/g_Pt_, respectively. Among the as-synthesized Au@Pt NPs, the ECSAs of the Au_95_@Pt_5_ NPs, Au_90_@Pt_10_ NPs, and Au_85_@Pt_15_ NPs are lower than that of commercial Pt/C (71.19 m^2^/g_Pt_). Under the self-supporting situation, for Au_99_@Pt_1_ NPs, a small quantity of small Pt NPs was distributed on the surface of the spiny Au NPs. Therefore, extensive agglomeration can be inhibited. In the Au_95_@Pt_5_ NPs, Au_90_@Pt_10_ NPs, and Au_85_@Pt_15_ NPs, small Pt NPs were overgrown on the surface of the spiny Au NPs, and the platinum layer thickened with the increase in H_2_PtCl_6_ concentrations, so the ECSA gradually decreased. This result is consistent with the SEM and TEM observations.

The electrocatalytic property of the Au@Pt NPs for the MOR was examined and compared with that of commercial Pt/C catalysts in a nitrogen-purged 0.5 M H_2_SO_4_ + 0.5 M methanol solution with a scan rate of 50 mV/s. Figures 4B,C display typical CV curves of the methanol oxidation on the Au@Pt NPs and commercial Pt/C catalysts, showing them to differ only slightly in shape, indicating a similar reaction pathway^72^. In the forward sweep, the anodic peak is observed at approximately 0.70 V, which could belong to the characteristic methanol oxidation on the electrode surface. An oxidation peak at approximately 0.45 V in the reverse scan is probably associated with the removal of the residual carbon species formed in the forward scan. Slight shifts were found in the mass activity CVs and specific activity CVs (Fig. 4B,C). During the forward scan, a slight positive shift compared to Pt/C occurred when the Pt loading increased to 10%, and a slight negative shift occurred when the loading reached 15%, which is associated with the loading amount of Pt on the spiny Au NPs and direct methanol oxidation^73, 74^. The slight positive shift in the reverse scan was caused by the decrease in Au oxidation, which is higher than that of Pt oxides^75^. Histograms of the mass activities and specific activities for the five catalysts at the peak current for the MOR were calculated and are shown in Fig. 4D,E, respectively. According to the peak current normalized against the ECSA, the Au_90_@Pt_10_ NPs has a specific activity of 0.68 mA/cm^2^, which is 4.6 times that of commercial Pt/C. In a previous report, the specific activities of Pt−Au nanoporous leaf and Pt−Au nanocrystal for the MOR are 1.8 and 3.4 times that of commercial Pt/C, respectively, less than the 4.6-fold increase in this work^61, 76^.

The mass activities of the Au@Pt NPs were higher than that of commercial Pt/C toward methanol oxidation, and the Au_95_@Pt_5_ NPs showed the best catalytic performance, 3.9 times that of commercial Pt/C. It is reasonable to deduce that the introduction of Au into Pt can increase the catalytic activity of the Pt. In contrast to the carbon support in commercial Pt/C, the improved catalytic activity of Au@Pt NPs can be ascribed to electronic effect modifications. As demonstrated by XPS, the binding energies of Pt in the Au@Pt NPs are up-shifted, which could decrease the CO adsorption energy on Pt and favor C-H cleavage on Pt sites owing to the shift of the d-band center^36, 77^. In addition, Au could advance the oxidation of CO to enhance the tolerance of CO. As a result, Au@Pt NPs could eliminate the CO intermediates more easily than commercial Pt/C could. On the other hand, unlike with the agglomeration of Pt NPs, the platinum layer on the Au@Pt NPs is thinner, adhering to the surface of the spiny Au NPs, and well dispersed, accordingly resulting in a high mass activity 3.9 times higher than that of commercial Pt/C, which is higher than previously reported values^31, 61, 78^.

The catalytic performances of the Au@Pt NPs shows that the amount of Pt on the surface of the spiny Au NPs has an optimal value, which has an important influence on the catalytic properties. As shown in Fig. 4D and E, the catalytic properties of the Au_95_@Pt_5_ NPs and Au_90_@Pt_10_ NPs were significantly better than those of the Au_99_@Pt_1_ NPs and Au_85_@Pt_15_ NPs. This result is similar to those reported in previous literature^61^, where the optimal MOR activity appears when 10% Pt is loaded on Au HNU surfaces. In our Au_85_@Pt_15_ NPs, all the Au metal is embedded in the center, and only the Pt NPs form the surface. Thus, Au metal is restricted to enhancing the catalytic properties, leading to catalytic abilities lower than those of the Au_95_@Pt_5_ NPs and Au_90_@Pt_10_ NPs. As demonstrated by TEM, the Pt NPs in the Au_99_@Pt_1_ NPs are rare on the surfaces of the spiny Au NPs. Therefore, the low Pt content on the surface of the Au_99_@Pt_1_ NPs would also reduce the catalytic properties for the MOR.

The steady-state catalytic activities of five catalysts were evaluated using chronoamperometry at a constant potential of 0.60 V for a period of 4000 s, and the resulting *i*-*t* curves are shown in Fig. 4F. The polarization currents for all the catalysts decrease rapidly because intermediate species are formed during the MOR process. The methanol oxidation current density of the Au_90_@Pt_10_ NPs over a period of 4000 s is the highest among those of all the catalysts examined. At the end of 4000 s, the Au_90_@Pt_10_ NPs display much slower current density decay with time, further revealing a better tolerance toward the CO-like intermediates.

## Conclusion

In summary, an economical, surfactant-free and efficient synthetic route using a seeded-growth method was used to form Au@Pt NPs. The Au@Pt NPs exhibited important improvements in catalytic response in comparison to commercial Pt/C catalyst towards methanol oxidation in acidic conditions. The best response for methanol oxidation was achieved from the Au_90_@Pt_10_ NPs, which was 4.6 times greater than that of commercial Pt/C in terms of per mg Pt activity in acidic media. The as-synthesized catalysts are not only very efficient in reducing the usage of Pt but are also promising candidates for methanol electro-oxidation.

## Methods

### Reagents and chemicals

Hydrogen hexachloroplatinate (IV) (H_2_PtCl_6_, ≥99.9%) and gold(III) tetrachloride trihydrate (HAuCl_4_·3H_2_O, ≥49.0%) were provided by Sigma. Commercial Pt/C (20 wt. %) was provided by Johnson Matthey. L-Ascorbic acid (AA, ≥99.7%) and methanol (≥99.7%) were purchased from Sinopharm Chemical Reagent Co. Ltd. All reagents and chemicals were used as received. Ultrapure water (18.2 MΩ cm) from a Milli-pore system was used in all experiments.

### Characterizations

X-ray photoelectron spectroscopy (XPS) was performed using a PHI 5000 Versa Probe with Al Kα as the excitation source. Scanning electron microscopy (SEM) images of as-prepared Au@Pt NPs were observed with a Carl Zeiss Supra 55 San electron microscope. Transmission electron microscopy (TEM) and HRTEM characterization were performed with an FEI Tecnai G2 F20 S-Twin electron microscope (200 kV). Structural analysis of the Au@Pt NPs was performed in scanning TEM (STEM) mode on an FEI Tecnai G2 F20 electron microscope (200 kV) equipped with an energy dispersive X-ray spectrometer (EDS). The STEM images were captured with a high-angle annular dark-field (HAADF) detector. Powder X-ray diffraction (XRD) was performed on a Rigaku D/max-rA X-ray diffractometer with graphite monochromatized CuKα radiation. The ICP (VISTA-MPX) was used to determine the content of the as-prepared Au@Pt NPs.

Measurement of the electrochemical properties was performed on a CHI-660D electrochemical workstation with a conventional three-electrode system (ChenHua Corp., Shanghai, China). An as-prepared Au@Pt NP-modified carbon glass electrode (GCE, 3 mm in diameter, 0.071 cm^2^) served as a working electrode. A Pt wire was used as the counter electrode, and an Ag/AgCl electrode was used as the reference electrode. A water suspension of the as-prepared Au@Pt NPs (6 µL, 5.872 × 10^−11^ mol) was dropped onto the cleaned GCE and dried in air at room temperature. After evaporation of the water, 5 µL of Nafion solution (0.05 wt. %, diluted from 5 wt. % Nafion with ethanol) was pipetted onto the catalyst film and dried in air. The blank scans were performed in 0.5 M H_2_SO_4_ purged with nitrogen and cycled from −0.2 to 1.1 V. Methanol oxidation reactions (MORs) were performed in a N_2_-saturated aqueous solution containing 0.5 M methanol and 0.5 M H_2_SO_4_. All measurements were recorded with a scan rate of 50 mV s^−1^. Chronoamperometric curves for the MOR were recorded at the potential of 0.60 V for 4000 s. All the potentials in this study were reported with respect to Ag/AgCl, and all electrochemical data were obtained at room temperature.

### Preparation of the spiny Au NPs

Five hundred microliters of gold seeds (40 nm) was added to aqueous solutions (approximately 6.5 pH) adjusted by 6 mL of sodium citrate solution (5%). Under mild shaking, 0.3 mL of 0.01 M HAuCl_4_ was added into the seeds mixture, shifting the color of the solution from pale pink to cyan, blue and light purple. The resulting spiny Au NPs were collected by centrifugation at 5000 rpm for 7 min, washed by water several times and were dispersed into water for further use.

### Preparation of the Au@Pt NPs

In a typical synthesis, a mixture of 30.00 mL of water and 1.00 mL of the spiny Au NP solution (7.34 nM) was added to a 50 mL glass bottle. Then, 1.00 mL of 0.1 M L-ascorbic acid was added into the above solution with magnetic stirring. After that, the bottle was placed into a water bath at 90 °C. Then, 0.2 mL of H_2_PtCl_6_ solution (20.0 mM) was added with strong stirring, and the solution was maintained at 90 °C for 15 min. Finally, the product was collected by centrifugation at 3000 rpm for 7 min and washed by water several times. The Au@Pt NPs with different Pt ratios (Au_99_@Pt_1_, Au_95_@Pt_5_, Au_90_@Pt_10_, Au_85_@Pt_15_) were synthesized by varying the injected volume of the Pt precursor from 0.1 to 0.4 mL.

## Electronic supplementary material

Supporting Information
