# Supplementary material for: A self-supporting bimetallic Au@Pt core-shell nanoparticle electrocatalyst for the synergistic enhancement of methanol oxidation
Source: Sci Rep. 2017 Jul 24;7:6347. doi: 10.1038/s41598-017-06639-5 (PMC5524951; doi:10.1038/s41598-017-06639-5)
Supplement: Supplementary file 1 — Supporting Information [file 41598_2017_6639_MOESM1_ESM.doc]

**Supporting Information**

**Free-supporting bimetallic Au@Pt core-shell nanoparticles electrocatalyst for synergetic enhancement of methanol oxidation**

Changhui Tan^1,2,3,+^, Yinghui Sun^4+^, Jianzhong Zheng^1^, Dan Wang^1^, Ziyang Li^1^, Huajie Zeng^1^, Jun Guo^5^, Liqiang Jing^3^, Lin Jiang^1^,*

Table S1. The amount and ratio of Au and Pt in Au@Pt NPs.

|  | Au_99_@Pt_1_ NPs | Au_95_@Pt_5_ NPs | Au_90_@Pt_10_ NPs | Au_85_@Pt_15_ NPs |
| --- | --- | --- | --- | --- |
| Au in Au@Pt NPs by ICP (µg) | 159.624 | 127.264 | 134.939 | 124.73 |
| Pt in Au@Pt NPs by ICP (µg) | 1.596 | 6.630 | 14.841 | 21.783 |
| Pt from added reagent (µg) | 6.240 | 12.48 | 18.72 | 24.96 |
| Ratio of Au in Au@Pt NPs | 99 | 95 | 90 | 85 |
| Ratio of Pt in Au@Pt NPs | 1 | 5 | 10 | 15 |


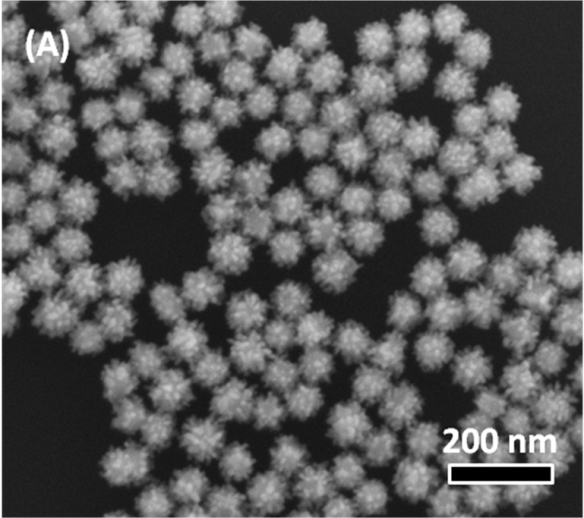

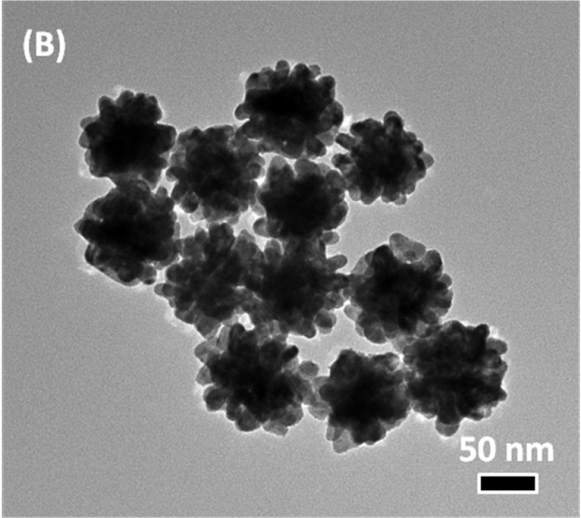


Figure S1. Morphology of spinal Au NPs (A) SEM and (B) TEM

Figure S2. HR-TEM of spinal Au NPs (A) and Au_90_@Pt_10_ NPs (B). Scale bare: 2 nm.

Figure S3. CV curves of spinal Au NPs in 0.5 M N_2_-saturated sulphuric acid solution and 0.5 M H_2_SO_4_ + 0.5 M CH_3_OH.
